# Considerations for Neuromorphic Supercomputing in Semiconducting and Superconducting Optoelectronic Hardware

**DOI:** 10.3389/fnins.2021.732368

**Published:** 2021-09-06

**Authors:** Bryce A. Primavera, Jeffrey M. Shainline

**Affiliations:** ^1^National Institute of Standards and Technology, Boulder, CO, United States; ^2^Department of Physics, University of Colorado Boulder, Boulder, CO, United States

**Keywords:** neuromorphic, superconducting electronics, optoelectronic, large-scale computing systems, spiking network, photonics

## Abstract

Any large-scale spiking neuromorphic system striving for complexity at the level of the human brain and beyond will need to be co-optimized for communication and computation. Such reasoning leads to the proposal for optoelectronic neuromorphic platforms that leverage the complementary properties of optics and electronics. Starting from the conjecture that future large-scale neuromorphic systems will utilize integrated photonics and fiber optics for communication in conjunction with analog electronics for computation, we consider two possible paths toward achieving this vision. The first is a semiconductor platform based on analog CMOS circuits and waveguide-integrated photodiodes. The second is a superconducting approach that utilizes Josephson junctions and waveguide-integrated superconducting single-photon detectors. We discuss available devices, assess scaling potential, and provide a list of key metrics and demonstrations for each platform. Both platforms hold potential, but their development will diverge in important respects. Semiconductor systems benefit from a robust fabrication ecosystem and can build on extensive progress made in purely electronic neuromorphic computing but will require III-V light source integration with electronics at an unprecedented scale, further advances in ultra-low capacitance photodiodes, and success from emerging memory technologies. Superconducting systems place near theoretically minimum burdens on light sources (a tremendous boon to one of the most speculative aspects of either platform) and provide new opportunities for integrated, high-endurance synaptic memory. However, superconducting optoelectronic systems will also contend with interfacing low-voltage electronic circuits to semiconductor light sources, the serial biasing of superconducting devices on an unprecedented scale, a less mature fabrication ecosystem, and cryogenic infrastructure.

## 1. Introduction

The foundations of cognition remain a great frontier of science, with potentially enormous ramifications for technology and society. A hardware capable of simulating spiking neural networks with the scale and complexity of the brain or even beyond could be a powerful tool in deciphering this enigma. Achieving such large-scale systems has proven to be non-trivial with established CMOS hardware (Furber, 2016). A significant challenge will be to enable efficient communication with low-latency amongst billions or trillions of neurons. Optics appears well-matched to the task, as the lack of resistive, capacitive, and inductive parasitics makes optical links more amenable to high fan-out than electrical interconnects (Shainline et al., 2019). While digital systems partially circumvent this issue by leveraging time-multiplexing to artificially increase fan-out (Young et al., 2019), multiplexing introduces latency that scales exponentially above a certain data load (Hennessy and Patterson, 2011). Optical interconnects may enable direct connections between neurons which would eliminate all traffic-induced delays and support larger, faster, and more interconnected networks. However, while the lack of interaction between photons is beneficial for reducing parasitics during communication, it is a detriment to computation. Electronic circuits are better suited to implement complex, nonlinear neuronal functions. It is reasonable to anticipate performance gains from optoelectronic neural systems leveraging optics for communication and electronics for computation, provided the hardware can be realized.

Our proposal to fabricate a direct, physical connection between every pair of connected neurons is known as the fully dedicated axon approach to communication (Segal et al., 2016). While this strategy requires largely fixing network topology in hardware—a chief disadvantage when compared with highly reconfigurable digital systems—the reduced overhead and elimination of communication bottlenecks will greatly benefit performance. We further specify that all synapses, dendrites, and neurons utilize fully dedicated electronic circuits, so that each element of hardware has a one-to-one correspondence with its information-processing role in the neural system. This fully dedicated approach is advantageous if one aspires to create a diverse array of synaptic and dendritic behaviors at each neuron, as observed in biological neural systems (Marder, 1987; Euler and Denk, 2001). For instance, a different time constant or plasticity mechanism could be implemented at every synapse on a single neuron. Perhaps more importantly, fully dedicated components eliminate the auxiliary hardware required to perform multiplexing operations. Further, performing synaptic weighting and temporal dynamics in the electronic domain allows for binary optical communication, which minimizes the amount of optical energy per spike and reduces noise incurred by communication. The scope of this paper is therefore limited to networks meeting these three conditions:

1. Direct, optical connections are utilized for communication between neurons (fully dedicated axons).
2. Optical communication is binary. The amplitude of the optical signal carries no information.
3. All synaptic, dendritic, and somatic computations are performed by fully dedicated electronic circuits.

**Figure 1:**
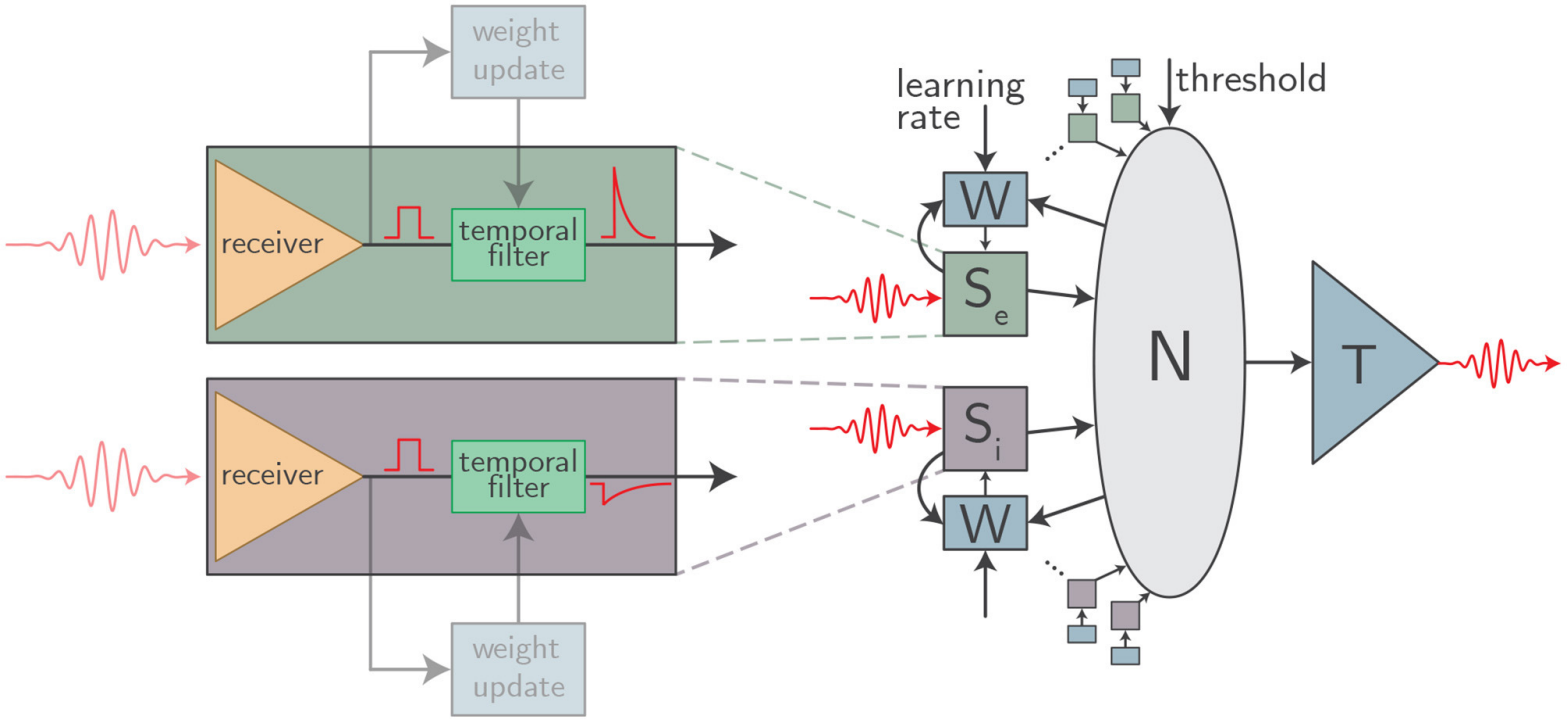
An abstract schematic of the class of optoelectronic neurons meeting our three criteria. Each synapse (*S*_*e*_ and *S*_*i*_ for expiatory and inhibitory synapses, respectively) is implemented with a physical circuit block containing a detector and a temporal filter. The detector produces an all-or-nothing electrical pulse upon receipt of an optical spike which is then processed by the filter. The parameters of the filter (time constant, weight, etc.) can be set individually for each synapse. A local weight update circuit (W) implements plasticity mechanisms at each synapse. Synaptic outputs are integrated in the soma (N) which drives an optical transmitter to downstream connections upon reaching threshold.

With these conjectures established, a picture of the hardware under consideration begins to emerge. There is a single optical transmitter at each neuron. This light emitter produces a short pulse of light each time the neuron spikes. The optical pulse is coupled into a waveguide, and optical power is tapped from the waveguide for each downstream synapse. Each synapse contains a photodetector which registers an all-or-nothing synapse event. From there, all synaptic weighting, spike-train filtering, dendritic processing, signal summation, neuronal thresholding, and plasticity mechanisms are implemented in the electronic domain with tailored integrated circuits. A schematic of this general framework is shown in Figure 1.

There are potentially multiple ways to physically implement this model. The remainder of this paper will discuss two possible implementations—a superconducting platform and a room-temperature all-semiconductor system. The superconducting platform, known as SOENs (Superconducting OptoElectronic Networks) is discussed in prior work (Shainline et al., 2017b, 2019; Shainline, 2019, 2021). In short, optical links are formed from semiconductor light sources and superconducting nanowire single photon detectors (SNSPDs). Computation is performed with analog Josephson junction (JJ) circuits and memory is implemented with persistent current in superconducting loops. The semiconductor implementation is imagined as an exact analog of the SOENs platform, except without the benefits (or limitations) of cryogenic elements. Traditional photodiodes enable optical communication, analog CMOS circuits provide computation, and emerging memory devices provide synaptic memory.

This paper seeks to analyze the suitability of both platforms for implementing large-scale optoelectronic neuromorphic networks. Despite limiting our discussion only to architectures meeting our three conjectures, there remains a vast space of design choices, making it difficult to draw hard-and-fast conclusions. Nevertheless, interesting guidelines can be obtained by analyzing limits of technologies most likely to be used in each platform. Important benchmarks for device performance are also identified, which may be of use in monitoring the development of this field.

## 2. Communication

### 2.1. Optical Receivers

We begin analysis of optical interconnects with receivers. There are two ways the receiver influences the power budget of an optical link: (1) The receiver (and the electrical components it must drive) sets the minimum optical signal that must be produced by the light source, and (2) the receiver may require electrical power of its own to run. It is found that the energy per spike may be quite similar in both platforms once cooling is accounted for in the superconducting case. However, the optical power required from light sources is reduced by a factor of 1,000 in the superconducting case, at least when compared to the semiconductor receivers of comparable total efficiency, which omit transimpedance amplifiers (Miller, 2017).

#### 2.1.1. Superconductor Receivers

**Figure 2:**
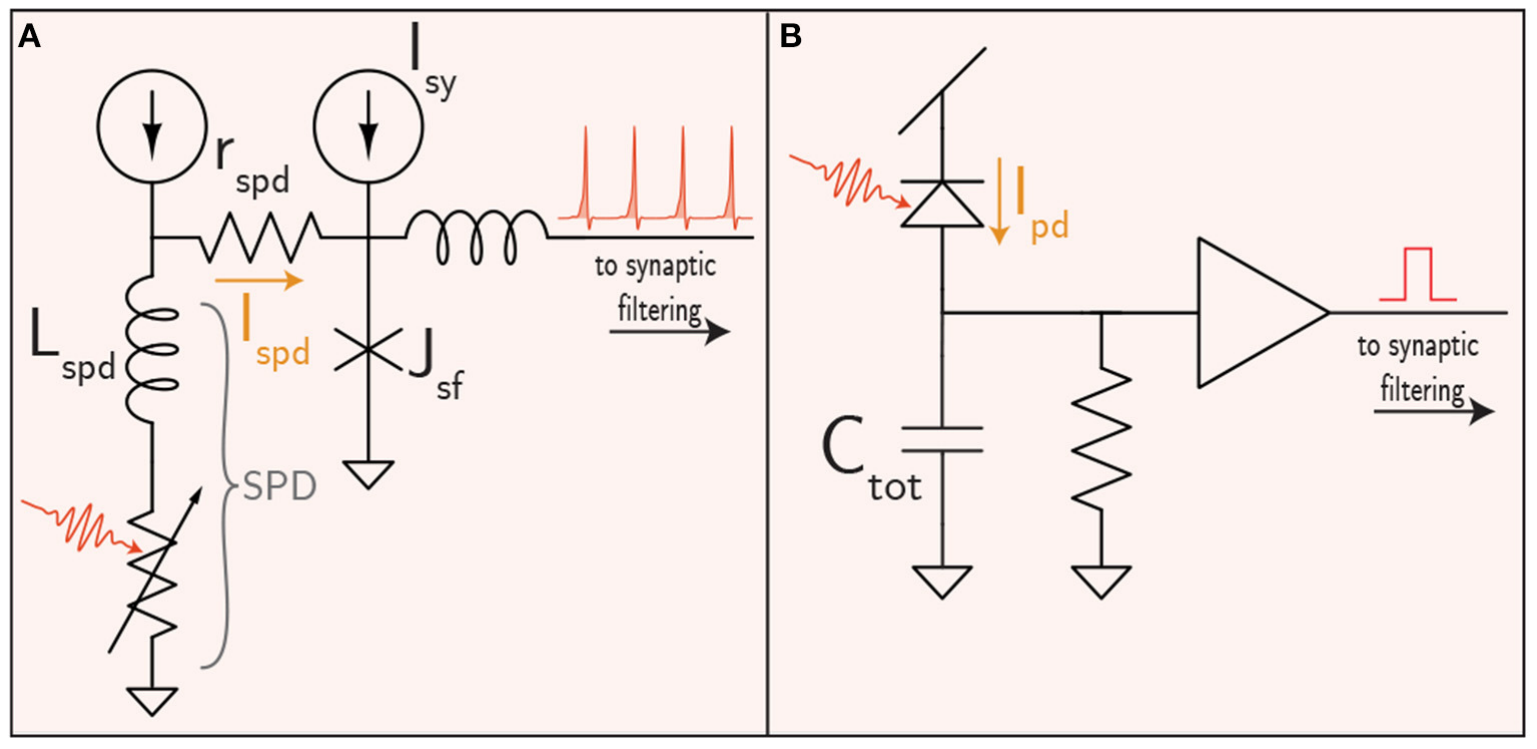
Receivers for the **(A)** superconducting and **(B)** semiconducting platforms. Note that synaptic weighting for the semiconductor case is included in the filtering circuitry, shown in Figure 4B.

As stated previously, the SOENs platform utilizes SNSPDs to detect optical signals as faint as a single photon. Physically, an SNSPD is a superconducting nanowire biased with a current source (*I*_spd_ ≈ 10 μA). The simple structure makes fabrication and waveguide integration straightforward (Sprengers et al., 2011; Pernice et al., 2012; Akhlaghi et al., 2015; Ferrari et al., 2015, 2018; Sahin et al., 2015; Shainline et al., 2017a; Buckley et al., 2020a). Photons traveling through a waveguide evanescently couple to a nanowire on the surface of the waveguide. A single photon has enough energy to drive the nanowire from the superconducting phase to a resistive state. In SOENs receivers, this momentarily redirects the bias current along an alternate conduction pathway that activates a JJ circuit to register the synapse event and conduct further synaptic processing (Figure 2A).

While an SNSPD itself dissipates zero static power, electrical power is still required for superconducting receivers. Current biases will require some power, but should be shared by many devices (section 3), ameliorating the cost. More important is dynamic electrical power consumption associated with detection events. The nanowire has an inductance, *L*_spd_, that stores energy from the current bias. During a detection event, this energy is dissipated in the resistor *r*_spd_. The electrical energy necessary to detect each photon is then $\frac{1}{2}L_{\text{spd}}I_{\text{spd}}^{2}$. *L*_spd_ can be as low as 100 nH, resulting in an electrical energy consumption (*E*_spd_) of around 5 aJ/spike.

Since an SNSPD is capable of detecting single photons, it will operate near the quantum limit of optical communication (Razavi, 2012). We assume that the detection of a single photon will be treated as the registering of a synaptic event. The probability of a light source producing a spike with a certain number of photons within a fixed time window is given by a Poisson distribution. We will also conservatively assume a detection efficiency η_*D*_ of 70% (higher detection efficiency is certainly possible Marsili et al., 2013; Reddy et al., 2020). The probability of measuring zero photons during a spiking event is then given by:

(1) $$\begin{array}{l} P\left(0\right)=\overset{\infty }{\underset{k=0}{\sum }}\frac{N_{ph}^{k}e^{-N_{ph}}}{k!}{\left(1-\eta_{D}\right)}^{k}=e^{-N_{ph}\eta_{D}}, \end{array}$$

where *N*_*ph*_ is the average number of photons per spiking event. Neural systems are known for remarkable robustness to and even utilization of noise (Stein et al., 2005; McDonnell and Ward, 2011). Detecting only 99% of spikes may be tolerable and would still represent a significant improvement over biology, wherein synapse reliability is typically in the range of 5–80% (Allen and Stevens, 1994; Lisman, 1997). From Equation (1), this would correspond to roughly 7 photons (0.9 aJ for λ = 1.5 μm) needed to reach the receiver. The total number of photons produced by the source will need to be higher to account for energy losses in the link. The total optical energy per spike, *E*_opt_, will be:

(2) $$\begin{array}{l} E_{\text{opt}}=\frac{N_{\text{ph}}h\nu }{\eta }. \end{array}$$

*hν* is the energy of a single photon and η is the total energy efficiency of the optical link. η includes all optical losses and the inefficiency of the transmitter. This efficiency factor will be highly dependent on the specifics of the platform, but for now we will leave it as a free variable. The total power consumed by the optical link is the sum of *E*_opt_ and *E*_spd_. Accepting a 1% error rate, these two contributions to the total energy will be roughly equal when η = 20%. Such a high efficiency is likely near the limits of physical possibility. For more realistic values of η, *E*_opt_ will dominate.

Importantly, superconducting electronics come with a cooling overhead (section 5). We conservatively assume that every watt of power produced at low temperature will require 1 kW of refrigeration power. System-level effective optical energy per spike for superconducting links will be no less than 1 fJ.

Fabrication of waveguide-integrated SNSPDs has become commonplace in recent years (Sprengers et al., 2011; Pernice et al., 2012; Akhlaghi et al., 2015; Ferrari et al., 2015, 2018; Sahin et al., 2015; Shainline et al., 2017a; Buckley et al., 2020a). SNSPD materials include NbN, NbTiN, WSi, and MoSi. Superconducting films (3–10 nm) can be sputtered at room temperature atop many substrates and patterned into wires from 50 to 5 μm wide using conventional lithography and etching. Multiple planes of SNSPDs have also been demonstrated (Verma et al., 2012)—a promising development for future large-scale neuromorphic systems (section 5). Waveguide-integrated NbN SNSPDs can reach photon count rates exceeding 1 GHz (Rosenberg et al., 2013; Vetter et al., 2016). However, slower detectors, such as MoSi and WSi SNSPDs with 20 MHz count rates, have demonstrated the best yields to date (99.7% Wollman et al., 2019). Previous statements that SOENs were limited to 20 MHz were motivated by these pragmatic concerns about the current state of fabrication (Shainline et al., 2019).

#### 2.1.2. Semiconductor Receivers

While semiconductor receivers are the predominant technology for long-distance optical communication, intra-chip optical receivers deviate significantly from their long-distance counterparts, as traditional transimpedance amplifiers likely consume too much electrical power, despite impressive optical sensitivities. This has led to the proposal of “receiverless” designs that omit amplifiers altogether (Miller, 2017). Receiverless communication uses a photodetector to directly drive the input of CMOS gates. Photons produce electron-hole pairs in the photodetector, which in turn charge the CMOS gate capcitance up to the switching voltage. A circuit diagram of the scheme is shown in Figure 2B in which a photodiode directly drives a CMOS digital buffer. A resistor is also placed in parallel to allow the receiver to reset. In principle the resistor is unnecessary if an optical reset is used as described in Debaes et al. (2003). The resistor would increase the minimum optical power necessary to register a spike and limit the bandwidth of the receiver.

With optical link efficiency η, the necessary optical energy required to drive the receiver to a voltage *V* is Miller (2017):

(3) $$\begin{array}{l} E_{\text{opt}}=\frac{C_{\text{tot}}V}{\eta R}. \end{array}$$

R is the responsivity of the detector, typically of order 1 A/W. *C*_tot_ includes the photodiode capacitance, the CMOS gate capacitance, and any wiring capacitance. It is reasonable to consider values for *C*_tot_ at the femtofarad level. For 1.5 μm photons and a required voltage swing of 0.8 V, *E*_opt_ ≈ 0.7 fJ (5000 photons) for unit efficiency. This is similar to the superconducting case, once cooling is considered. If two optical communications links were identical in all measures (source efficiency, optical losses, etc.) except one was cooled to 4 K with SNSPDs and the other operated at room-temperature with photodiodes, then communicating a spike would cost nearly the same energy at the system level in each link. The power required for cryogenic cooling pays for itself with reduced light levels in the optical link. Cooling semiconductor receivers to 4 K does not appreciably improve the situation, as the number of photons required in the receiverless case is related to charge, capacitance, and voltage, not thermal noise. For capacitances below 1 fF (a difficult task), semiconductor receivers could potentially consume even less energy than their superconducting counterparts. Waveguide-integrated femtofarad photodiodes have been demonstrated in both SiGe and Ge (DeRose et al., 2011). Polysilicon photodiodes are also attractive for increased manufacturability Mehta et al. (2014). Most photodiodes have far better speed than required for neuromorphic applications, reaching up to 45 GHz (DeRose et al., 2011).

Just as with SNSPDs, semiconductor receivers will also require electrical power, even if it is minimized by the receiverless approach. In this case, there will be static power dissipation through the leakage current of the photodiode. Assuming a 1 V bias, a leakage current on the order of 1 nA (Zhang et al., 2020a), and an optical link efficiency of 1%, this static dissipation would dominate power consumption for average spiking rates below 10 kHz. The development of low capacitance, zero-bias photodiodes (Nozaki et al., 2018) would be a major advantage toward making efficient, low frequency networks. Static power consumption is also a major question for many avalanche photodiode (APD) receivers. Avalanche gain could provide a significant (at least one order of magnitude) reduction in the necessary optical power per spike (Miller, 2017). While often associated with higher bias voltages, germanium waveguide-integrated avalanche detectors have been demonstrated to provide 10 dB of gain even at 1.5 V bias (Assefa et al., 2010). However, dark current is still typically in the microamp range for such detectors (Assefa et al., 2010; Virot et al., 2014), meaning that brain-scale networks are likely out of reach due to power constraints (section 5). APDs may be of interest in smaller, faster spiking networks, however. Another intriguing possibility is to reduce static power consumption through cooling, as the dark current could potentially be reduced by orders of magnitude (Pizzone et al., 2020). However, in that case one forfeits a major advantage of the semiconductor approach.

**Figure 3:**
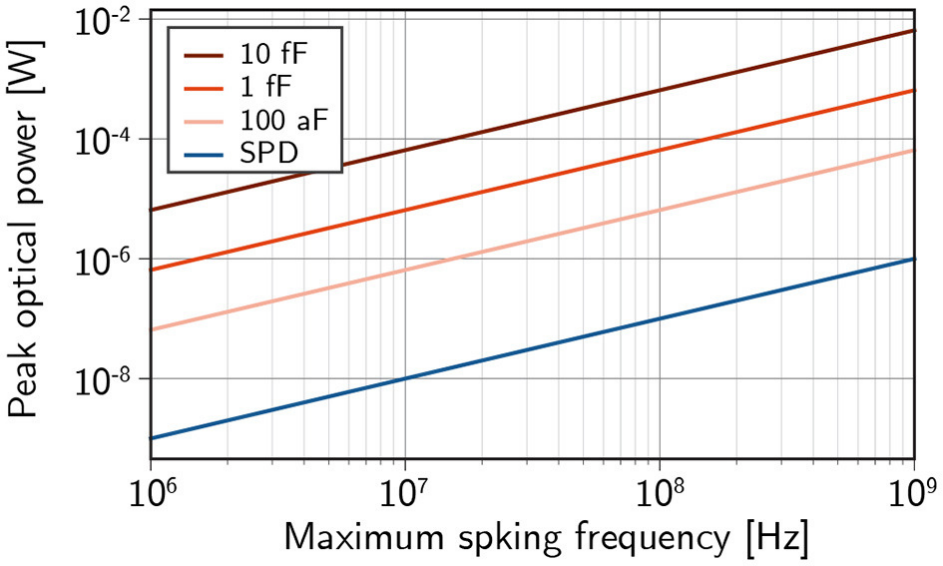
The required optical power to drive 10^3^ downstream synapses within one inter-spike interval for a given spiking frequency assuming receiverless photodiodes with optical link efficiency η = 1.

While the receiverless scheme is promising for achieving low energies per spike, it places significant burden on the transmitter side of the link. Neuromorphic applications magnify this burden, as neurons are expected to drive thousands of downstream connections in parallel. Additionally, the receiver capacitance must be charged quickly to maintain high spiking frequencies. The result is that relatively large optical power is required from transmitters. The best case (η = 1) scenario is shown in Figure 3. Semiconductor receivers can be expected to require around one thousand times the optical power of superconducting receivers and the highest spiking frequency of a neuron could very well be limited by the power output of the light source. The ramifications of this result on prospective light sources are discussed in the next section.

### 2.2. Optical Transmitters

The transmitter is expected to dominate the power budget of optical links for both platforms. Room-temperature, CMOS-integrated light sources have been a holy grail for decades, but materials integration issues have kept this prized objective out of reach. For superconducting systems, SNSPDs drastically lower the power requirements of light sources, while cryogenic temperatures improve light source efficiency. Light sources are likely significantly simpler in the superconducting case. However, interfacing low-voltage superconducting electronics with semiconductor light sources (McCaughan et al., 2019) presents an obstacle that is absent from the all-semiconductor platform.

#### 2.2.1. Integrated Light Sources

Optical coherence is not a requirement for the envisioned system. NanoLEDs are thus an attractive option due to their ease of fabrication, lack of threshold current, and improving efficiency with shrinking scale (Romeira and Fiore, 2019). However, nanoLEDs struggle to produce optical power significantly greater than 1 μW Romeira and Fiore (2019). While semiconductor systems targeting spiking frequencies in excess of 1 MHz may be forced to turn to lasing, nanoLEDs should be more than sufficient for superconducting platforms. Either way, integrating millions of light sources on a 300 mm wafer remains highly challenging. The indirect band gap of silicon drastically reduces light emission. Off-chip light sources are used in some applications, but are likely untenable for massive systems, as their high static power consumption is incommensurate with the sparsity of neural activity. Integrated light sources would be a tremendous boon, if not a requirement for the success of large-scale optoelectronic neuromorphic computing. There are two courses of action: (1) force silicon to emit light through either material and/or environmental modifications or (2) integrate direct bandgap materials on silicon.

Many strategies toward silicon light sources have been pursued (Iyer and Xie, 1993; Shainline and Xu, 2007) including quantum confinement in Si-based superlattices (Warga et al., 2008) and nanocrystals (Walters et al., 2005), emission from embedded erbium (Ennen et al., 1985; Palm et al., 1996), point-defect emitters (Brown and Hall, 1986; Bradfield et al., 1989; Bao et al., 2007; Rotem et al., 2007), extended defects (Ng et al., 2001), strain dislocations (Kveder et al., 2004), and engineering of the local density of optical states (Green et al., 2001). Total efficiency from 0.1% (Kveder et al., 2004) to 1% (Green et al., 2001) has been demonstrated at room temperature, but not at powers and areas suitable for the semiconductor receivers introduced in the previous section.

Abandoning silicon as an active optical element, many researchers turned toward epitaxial germanium grown on Si (Sun et al., 2009c). Like silicon, germanium is an indirect-gap semiconductor. However, the direct gap is only 136 meV higher than the indirect gap, and clever implementation of strain (Ishikawa et al., 2003; Ghrib et al., 2012; Tani et al., 2021) and heavy *n*-type doping (Liu et al., 2007; El Kurdi et al., 2009; Sun et al., 2009a; Camacho-Aguilera et al., 2013; Virgilio et al., 2013) can lead to appreciable direct, radiative recombination. These efforts have led to Ge-on-Si lasers (Sun et al., 2009b; Liu et al., 2010), but it has proven difficult to reduce the threshold current and increase device efficiency. Another approach is to grow SiGe with a hexagonal lattice on GaAs, leading to a direct gap (Fadaly et al., 2020), but this does little to solve integration problems.

At present, neither Si nor Ge emission has proved satisfactory for the needs of digital communication, so integrating III-V materials on silicon substrates has received significant attention. Pending a watershed moment in silicon sources, III-V integration will be required for the semiconductor platform (although not necessarily in the superconductor case, where low-temperature changes the physical context). Epitaxial growth would be an attractive solution for III-V integration due to the high throughput (Norman et al., 2018), but defects due to lattice mismatch have so far prevented this method from large-scale adoption. III-V quantum dots are more robust to such defects and have demonstrated high optical powers with small footprints (Chen et al., 2016; Jung et al., 2017; Norman et al., 2018), albeit typically grown on offcut Si substrates that are not CMOS compatible or with thick buffer layers that make optoelectronic contact difficult. More work is required to realize scalable, cost-effective integration of III-V quantum dot light sources with CMOS electronics, passive photonic waveguides, and efficient photodetectors. Without epitaxial growth, the semiconductor platform would be less scalable due to the limited size of III-V wafers and the expense of performing wafer bonding. A variety of schemes have been proposed (Norman et al., 2018; Tang et al., 2019), including die-level bonding (Song et al., 2016; Crosnier et al., 2017), wafer-level bonding (Hu et al., 2019; Szelag et al., 2019; Jiao et al., 2020), transfer printing (Justice et al., 2012; Zhang et al., 2018a, 2019), and selective-area epitaxy (Han et al., 2021), but these approaches still appear cumbersome when seeking the scale of integration considered here.

The situation is significantly more favorable for cryogenic systems. Low temperature often reduces non-radiative recombination (Sandiford, 1958; Gurioli et al., 1991; Dolores-Calzadilla et al., 2017), improving efficiency for both silicon and III-V light sources. The case of Ge at low temperature is more subtle due to the pecularities of the pseudo-direct gap and inter-valley scattering that is more prevalent at higher temperatures (Sun et al., 2009c). The benefits are further compounded by the low optical power requirements of SNSPDs. When integrating III-V light sources with CMOS, the light sources must be integrated on top of the electronics after the high-temperature dopant activation steps have been performed. Superconductor electronics have no such high-temperature processing steps, so the light sources can be produced on a Si wafer before the electronics are realized. Problems related to offcut Si wafers and thick buffer layers are eliminated. Additionally, silicon light sources, with their superior potential for integration, demand exploration with the superconducting platform. Several silicon point defects typically quenched at room-temperature emerge as narrow-linewidth candidates for light sources in the telecommunications band (Davies, 1989; Sumikura et al., 2014; Buckley et al., 2017; Beaufils et al., 2018; Chartrand et al., 2018). While single-photon emission (Bergeron et al., 2020; Hollenback et al., 2020; Redjem et al., 2020) is not the objective in the present context, the narrow linewidth is also attractive for further efficiency gains via the Purcell Effect (Romeira and Fiore, 2018). LEDs have already been demonstrated with the W-center defect (Bao et al., 2007; Buckley et al., 2017), albeit with poor (10^−6^) efficiencies, limited by electrical injection efficiency rather than emitter lifetime. Photoluminescence studies are promising for orders of magnitude improvement (Buckley et al., 2020b), but more work is required to improve emission efficiency in an integrated-circuit context. If cryogenic silicon light sources become viable, the superconducting platform might hold a major scalability advantage over the semiconducting analog.

#### 2.2.2. Driving Circuitry

Both platforms require neurons to drive semiconductor light sources. The transmitter circuitry is thereby required to produce voltages on the scale of the bandgap of the optical source (≈ 1 V). CMOS circuitry, itself a semiconducting technology, naturally operates on this voltage, rendering the driving circuitry a non-issue. Standard MOSFET LED or modulator driving circuits (Halbritter et al., 2014; Bowers et al., 2016) can be straightforwardly adapted for neuromorphic applications. Superconductors, however, operate in an entirely different regime, with signals usually on the order of the superconducting energy gap (≈ 1 mV). The optimal method for interfacing superconducting electronics with semiconductor devices is still an area of active research. Recent progress has been made with devices utilizing the massive change in impedance during a phase transition between superconducting and resistive states. In McCaughan et al. (2019), a resistive element was heated using 50 mV pulses to thermally trigger a transition in a superconducting meander. The meander transitioned to a state with resistance in excess of 10 MΩ and was used to drive a cryogenic silicon light source waveguide-coupled to an SNSPD. While these results are promising, the light source was only pulsed at 10 kHz (due to poor source efficiency) and was fabricated on a separate chip. More work is needed to improve the speed, efficiency, and to monolithically integrate driving circuitry with LEDs.

## 3. Electronic Neuronal Computation

Electronic circuitry capable of performing neuronal dynamical operations will also be necessary. Biological neurons are increasingly recognized as sophisticated computational units (Koch and Segev, 2000; Stuart and Spruston, 2015; Hawkins and Ahmad, 2016; Sardi et al., 2017). Emulating such complicated behavior has been the subject of extensive investigation in both semiconducting (Vogelstein et al., 2007; Indiveri et al., 2011; Brink et al., 2013; Pfeil et al., 2013; Benjamin et al., 2014; Abu-Hassan et al., 2019) and superconducting platforms (Crotty et al., 2010; Shainline, 2019; Toomey et al., 2019). We do not attempt a comprehensive review of circuitry, but rather draw attention to issues specific to optoelectronic networks in both cases.

### 3.1. Semiconductor Electronics

The maturity of CMOS processing has allowed great strides in neuromorphic computing. While optical communication would likely also be advantageous in digital approaches, we focus on analog CMOS neurons for their perceived efficiency advantages (Mead, 1990; Rajendran et al., 2012). At a basic level, a neuron must perform three mathematical functions: summation of synaptic inputs, temporal filtering, and threshold detection leading to action potential generation. Summation can be achieved by exploiting Kirchoff's current law. Filtering can be implemented with elementary resistor-capacitor circuits. Thresholding is a natural function of transistors. Building upon this basic mapping, analog neurons have demonstrated a litany of biologically-inspired models (Indiveri et al., 2011; Liu et al., 2015).

It was found in the previous section that optical communication requires a minimum of about 1 fJ of energy to deliver a spike signal to each synapse. For realistic optical link efficiencies, this value will be at least an order of magnitude larger. Synaptic processing circuits would therefore ideally operate with an energy budget of 10–100 fJ to process a single spike. Somatic computation could comfortably consume power larger than that of synaptic processing by a factor of the average fan-out (perhaps 1,000). Many low-energy neuromorphic demonstrations are promising for reaching these targets. By reducing the membrane capacitance and supply voltage, a neuron capable of 25 kHz spike rates was demonstrated to consume only 4 fJ/spike (Sourikopoulos et al., 2017). Many other analog neurons, with energies ranging from femtoJoules to picoJoules per spike, fall comfortably below the power consumption of optical communication (Indiveri and Sandamirskaya, 2019). However, it remains to be seen if more complicated neurons and synapses, implementing a critical subset of behavior necessary for cognition, will be able to maintain such low power operation. In terms of speed, CMOS neurons have demonstrated spike rates in excess of 100 MHz (Schemmel et al., 2017). Optical communication should face few issues achieving such speeds, *if* sufficiently bright light sources can be efficiently integrated with CMOS circuits.

One challenge for the CMOS approach has been to design compact circuits with long time constants. Long time constants are important for systems targeting biological time scales (upwards of 500 ms) (Indiveri and Sandamirskaya, 2019) or power-law distributions of timescales to implement critical behavior (Beggs, 2007). Subthreshold transistor circuits operating with currents in the femtoamp to picoamp range minimize the size of capacitor needed to implement a specific time constant (Indiveri et al., 2011). The area constraints of this scheme are discussed in Supplementary Information A and compared to the superconducting approach.

**Figure 4:**
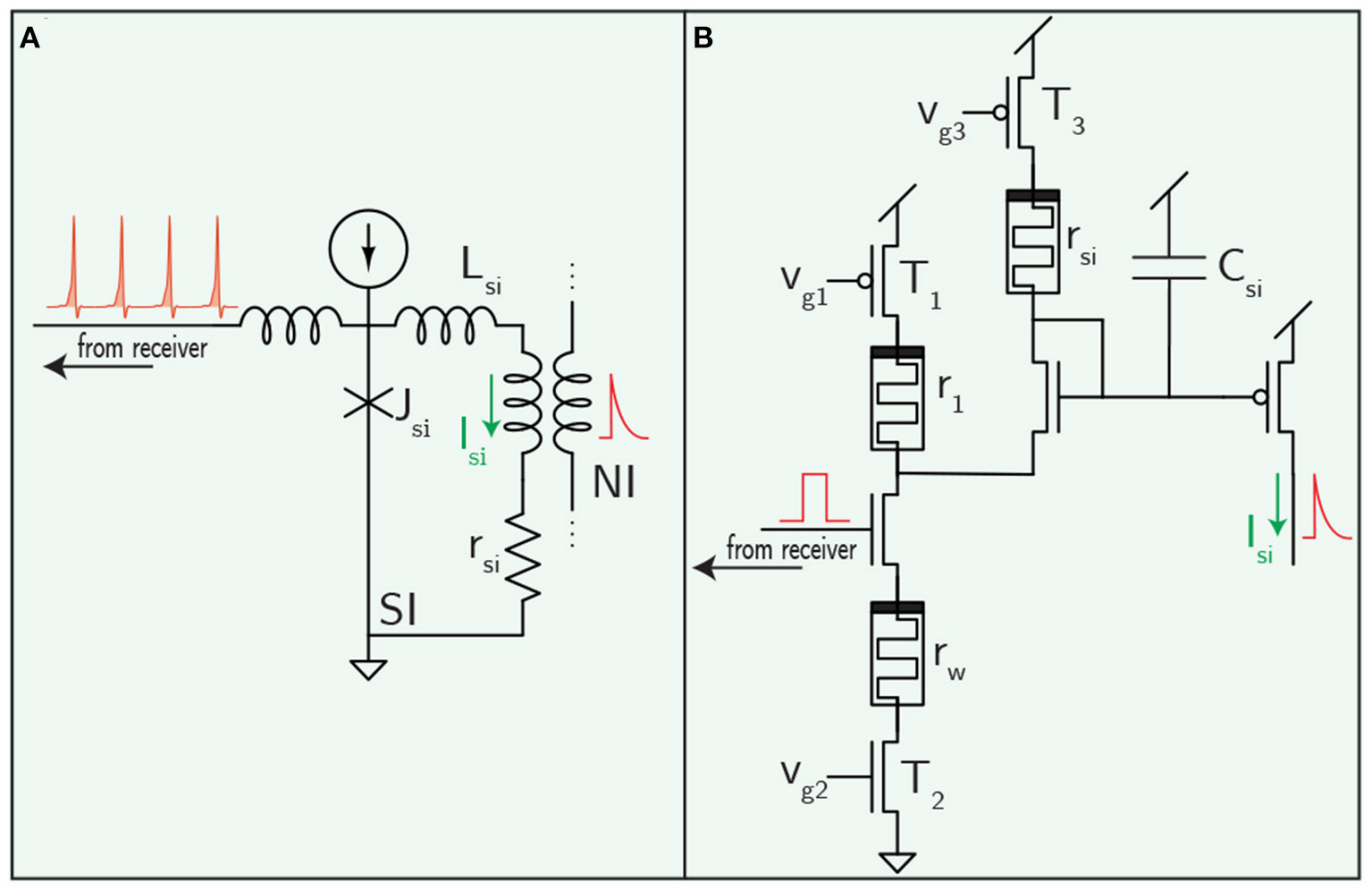
Synaptic filtering circuits for the superconductor **(A)** and semiconductor **(B)** cases. Weighting in the superconducting case was shown in Figure 2. The memristor-integrated DPI circuit pictured here is introduced in Ref. (Dalgaty et al., 2019).

For a concrete example, a circuit diagram for a memristor implementation of the popular differential-pair integrator (DPI) synapse is shown in Figure 4B (Dalgaty et al., 2019). The DPI produces a decaying exponential post synaptic signal in response to an input voltage pulse—potentially from an optical receiver. This leaky integrator behavior is characterized by a time constant set by the value of the filtering capacitance and the rate of leakage off the capacitor (Chicca et al., 2014). The time constant could potentially be programmed using memristors—an advantage over superconducting circuits that have been proposed to date.

### 3.2. Superconducting Electronics

Superconducting neurons have been studied nearly as long as CMOS implementations, with a mapping between neuronal functions and superconducting electronics identified in the early 1990s (Harada and Goto, 1991; Hidaka and Akers, 1991). In this case, Faraday's Law, governing the addition of magnetic flux through mutual inductors to superconducting loops provides the necessary synaptic summation function. Filtering is achieved through resistor-inductor blocks (or RC circuits in some cases Crotty et al., 2010). Josephson junctions (JJs) provide the requisite nonlinear thresholding element.

Like their CMOS counterparts, many superconducting circuits have now been designed to implement sophisticated neuronal dynamics. Superconducting neuromorphic circuits have been designed to implement a variety of bio-inspired neuron models (Crotty et al., 2010; Schneider et al., 2018a; Toomey et al., 2019), dendritic processing (Shainline, 2019), and have performed image classification in simulation (Schneider et al., 2017). The natural spiking behavior of JJs may even require a lower device count than analogous CMOS circuits for various leaky-integrate-and-fire models (Crotty et al., 2010). In short, it does not appear that superconducting circuits are any less capable of complex neuronal computation than CMOS, although experimental demonstrations lag far behind.

Superconducting electronics has long been pursued for gains in energy efficiency and speed. Indeed, superconducting elements dissipate zero static power and spike energies are frequently reported in the sub-femtojoule range, including refrigeration. Optical communication is likely to dominate power consumption for superconducting optoelectronic systems (Supplementary Information B). In terms of speed, fully electronic superconducting neurons may be capable of spike rates up to 100 GHz (Schneider et al., 2017, 2018a). However, this is orders of magnitude faster than any SNSPD can respond. This speed disparity is a notable difference between the superconducting and semiconducting architectures. While optical communication could be integrated with CMOS neurons with no degradation in speed, optoelectronic superconducting systems will likely be significantly slower than their fully electronic counterparts. This may be the cost of highly connected systems. That said, the extraordinary switching speed of JJs is still leveraged in optoelectronic networks to perform analog computations within synapses, dendrites, and neurons.

The ability of superconducting electronics to go slow might be just as compelling as their ability to go fast. While it can be challenging to implement long, biologically realistic time constants in CMOS neurons, superconducting loops can create time constants orders of magnitude higher than biology by adjusting the *L*/*R* ratio in synaptic and neuronal loops (See Figure 4A and Supplementary Information A). The ability to generate dynamics across many orders of magnitude in time also dovetails nicely with suggestions that critical behavior is important for cognition (Cocchi et al., 2017).

Fan-in has traditionally been considered a liability of superconducting electronics. If this were the case, it would clearly be an impediment to mature superconducting neuromorphic systems. For superconducting neurons designed to use single fluxons as synaptic signals, fan-in has recently been analyzed (Schneider and Segall, 2020), and it has been found that if a single synapse must be able to drive a neuron above threshold, fan-in may be limited to around 100. However, it is often not necessary for each synapse to be able to trigger a neuronal spike event. It has been analyzed elsewhere that if analog signals containing multitudes of fluxons are communicated from synapses to the neuron cell body, fan-in can likely scale to biological levels through the use of mutual inductors (Shainline et al., 2019). Using more fluxons comes with larger power consumption, but for optoelectronic systems, light production will likely still dominate.

While most diagrams of superconducting circuits (including those here) show many separate biases delivering current to various elements, the ability to construct circuits that can be biased in series will be critical to the scalability of this hardware. A separate bias for every synapse would be untenable in large-scale systems (Tolpygo, 2016). This mimics the evolution that occurred in superconducting digital electronics, in which the field has turned away from parallel biasing schemes and embraced serially biased platforms (Tolpygo, 2016) and current recycling schemes (Kirichenko et al., 2011). SOENs are potenially amenable to serial biasing, but this important point demands further analysis.

A superconducting synaptic filtering circuit is shown in Figure 4A. Synaptic weighting is implemented in the receiver circuit (Figure 2A), so this circuit block is only responsible for converting a train of fluxons into a decaying exponential post-synaptic potential reminiscent of biological and CMOS synapses. A resistor, *r*_si_, converts a superconducting persistent current loop into a leaky-integrator in a similar manner to the DPI synapse. The time constant is set by *L*_si_/*r*_si_, and the synaptic current can be added to a neuronal circuit through mutual inductors. Unlike the DPI synapse, this circuit does not have a programmable time constant, but does hold the potential to implement a wide range of different time constants by fabricating different values of *L*_si_ and *r*_si_.

## 4. Synaptic Memory

It has been apparent to the neuromorphic community for some time that large-scale neural systems will require innovative approaches to synaptic memory. A local, analog memory element unique to every synapse will provide the most efficient performance by eliminating memory retrieval and digital conversion. Important metrics for analog synaptic memory technologies include weight precision, volatility, area, write energy, write speed, and endurance (the effective number of cycles in a device's lifetime). We attempt to provide desired benchmarks for a few of these metrics in the specific case of optoelectronic networks. For this section, we assume a speedup of about 10^4^ over biology, for an average spike rate of 10 kHz and a maximum of 10 MHz. This is commensurate with both the maximum count rates of high-yield SNSPDs and some of the fastest CMOS electronic neuromorphic systems built to-date.

### 4.1. Memory Benchmarks

#### 4.1.1. Endurance

Large-scale neural systems require significant investments in money and time. Operational lifetimes on the scale of decades (10^9^ s), if not longer, are therefore essential. Such systems will be expected to learn continually during that lifespan, placing significant requirements on the durability of memory technologies. The number of times a synapse is updated in its lifetime is a function of neuron spiking frequency (*f*) and the number of synapses that are typically updated after each post-synaptic spike. Neuroscientific evidence has been presented that the number of active presynaptic inputs required to trigger a postsynaptic spike goes as $\sqrt{N}$, where *N* is the fan-in of the neuron—exceeding 1,000 for brain-like systems (van Vreeswijk and Sompolinsky, 1996; Vogels et al., 2005). We assume all synapses that contributed to the spiking of the post-synaptic neuron are updated with each spike. We then estimate the number of weight updates (*N*_update_) in the synapses's lifetime (*L*) will be:

(4) $$\begin{array}{l} N_{\text{update}}=\frac{Lf}{\sqrt{N}} \end{array}$$

For a decades-long lifetime, and a mean spiking frequency of 10 kHz, the total number of weight updates will be 10^11^. This is a challenging demand for many emerging non-volatile memory technologies.

#### 4.1.2. Update Energy

One would like the power dedicated to weight updates not to exceed the power used for optical communication. Once again invoking the assumption that $\sqrt{N}$ synapses are updated with each postsynaptic spike, we arrive at the following relation between the energy to produce a single spike (*E*_opt_) and that to update a single weight (*E*_update_):

(5) $$\begin{array}{l} E_{\text{update}}<\sqrt{N}E_{\text{opt}} \end{array}$$

Using the analysis in section 2, 1 fJ of energy needs to be delivered to the receiver in either platform. Assuming a transmitter efficiency of 1%, this would mean *E*_opt_ is 100 fJ. Therefore, for a fan-in of 1,000 synapses, *E*_update_ would ideally be no more than about 3 pJ. This value includes any energy consumption of peripheral circuitry, both static and that associated with programming. This efficiency appears to have already been met by several emerging memory technologies (Schneider et al., 2018b; Zahoor et al., 2020).

#### 4.1.3. Update Speed

An ideal system would be capable of implementing synaptic updates within the minimum inter-spike interval. While semiconductor optoelectronic systems could potentially produce spike rates in excess of 10 GHz (assuming sufficiently bright, integrated light sources can be achieved), synapses might need to be taken offline during WRITE operations, as it is unlikely that sophisticated plasticity mechanisms can be implemented in under 100 ps. Lower maximum frequencies would allow plasticity to be implemented without ever neglecting a spiking event. For our 10 MHz target, we desire memory updates in under 100 ns. Slower updates may not be completely intolerable, if network dynamics are robust to missed spikes during synaptic updates or to synaptic weights that are in the process of being altered.

#### 4.1.4. Weight Precision

The necessary weight precision will be determined by the specifics of a chosen learning model and the desired application. Weight precision has been the subject of much discussion. It has been suggested that 4-bit precision is sufficient for state-of-the-art mixed signal neuromorphic systems (Pfeil et al., 2012). Deep learning systems have also demonstrated success with 8-bit precision—a significant reduction from 32-bit floating point numbers (Wang et al., 2018). Hippocampal synapses in rats have been inferred to allow at least 26 different states (≈ 5 bit), which squares nicely with computer science findings (Bartol et al., 2015). It has also been argued that metaplasticity mechanisms are more important for lifelong learning than the bit-depth of the synapse (Fusi et al., 2005; Fusi and Abbott, 2007).

Target values for these key synaptic memory metrics are summarized in Table 1.

**Table 1:** List of desired performance metrics for synaptic memory in a system with average fan-out of 1,000, maximum spike rate of 10 MHz, average spike rate of 10 kHz, and spike energy of 100 fJ.

**Metric**	**Goal**
Endurance	>10^11^ updates
Update Energy	<3 pJ
Update Speed	<100 ns
Weight Precision	4-8 bits

#### 4.1.5. Programming Signals

One important criterion that eludes quantitative benchmarking is the complexity of programming circuitry for synaptic memory. Significant infrastructure for producing programming signals could limit scalability. For example, floating-gate synapses often require programming signals at significantly higher voltages than are likely to be used in other parts of the network. For large-scale systems, memories with simple programming requirements will be at an advantage. Superconducting loop memory (section 4.2.4) is intriguing from this standpoint, as the plasticity circuits operate with nearly identical signals and circuit blocks as those found in the rest of the network.

### 4.2. Proposed Technologies

#### 4.2.1. Room-Temperature Analog Memories

Many technologies have been proposed to implement synaptic weighting for room-temperature neuromorphic hardware, each with strengths and weaknesses (Upadhyay et al., 2019). The quest to find a suitable device for local synaptic memory dates back to the origins of the field, when Mead and colleagues investigated floating gate transistors (Diorio et al., 1998). Since then, floating gate synapses have been used to implement STDP (Ramakrishnan et al., 2011), are attractive as a mature alternative to emerging devices, and have been proposed for use in large-scale systems (Hasler and Marr, 2013). However, there are concerns about high programming voltages, speed, and endurance that may limit floating-gate memories to situations with less-frequent updates. More recently, momentum has shifted to other technologies (Zahoor et al., 2020). Memristive devices (Strukov et al., 2008; Yang et al., 2012; Abraham, 2018), commonly used in resistive random-access memory have emerged as a popular alternative, with recent demonstrations including monolithic integration with CMOS (Yin et al., 2019) and unsupervised pattern recognition with a simple network of synapses (Ielmini, 2018). Questions remain about high variability (both cycle-to-cycle and device-to-device) (Dalgaty et al., 2019), linearity, and endurance (Zahoor et al., 2020). Phase-change memory is another option, with its own demonstration of STDP (Ambrogio et al., 2016). Thermal management and endurance have been raised as issues (Upadhyay et al., 2019; Zahoor et al., 2020). Ferroelectric transistors present another alternative, as they have low variability, good potential for CMOS integration, and linearity (Kim and Lee, 2019). Spin-torque memory, 2D materials, and organic electronics have also been proposed as solutions. Interested readers should consult one of the many review articles on this topic (Kim et al., 2018; Upadhyay et al., 2019; Zhang et al., 2020b). The field is burgeoning with new devices for synaptic memory, but to-date none has been dominant enough to monopolize research. To our knowledge, no technology has been able to simultaneously meet the targets in Table 1, but progress in this area is encouraging.

#### 4.2.2. Superconducting Technologies

Many of the aforementioned technologies may also apply to superconducting optoelectronic systems, but their cryogenic operation has been scarcely explored. Two other types of memory, only accessible at low temperatures, have received the most attention for superconducting systems: magnetic Josephson junctions (MJJs) and superconducting loop memories. An important distinction from room-temperature technologies is that for superconducting memory to be truly non-volatile, it must retain its state both in the absence of a power supply and upon warming to room-temperature.

#### 4.2.3. Magnetic Josepson Junctions

MJJs have been proposed as a (nearly) non-volatile memory technology for superconducting neuromorphic computing. A two-terminal device, the critical current of an MJJ can be programmed by changing the magnetic order of a ferromagnetic material placed in the tunneling barrier of a JJ (Schneider et al., 2018b). MJJs are non-volatile with respect to electrical power, and there is optimism they can be made to retain their memory through a warm-up to room-temperature. Additionally, they provide remarkable performance with respect to the metrics given in Table 1. The energy per update is on the order of femtojoules (including cooling overhead), switching speeds are commensurate with firing rates exceeding 100 GHz, and devices can be scaled to tens of nanometers. All of these metrics surpass the requirements for optoelectronic networks, and can be exploited in all-electronic superconducting networks as well (Schneider et al., 2018a). More work is needed to analyze the scaling potential of MJJs with respect to yield. The magnetic fields used during programming can be produced with magnetic control lines, but spin-torque mechanisms may provide a more scalable solution. Finding an efficient, scalable solution to programming MJJs in large-scale systems thus remains an area of research that will be critical to their potential for adoption.

#### 4.2.4. Loop Memory

Superconducting loop memories have been in use for decades by the superconducting electronics community (Duzer and Turner, 1998; Kadin, 1999), but are not ideal for dense memory arrays commonly utilized as RAM in digital computing due to area concerns. In the case of optoelectronic spiking neural systems considered here, the objective is not to produce large RAM arrays, and size as well as addressing challenges do not emerge as significant impediments. Therefore, straightforward extensions of binary loop memories are the synaptic memory technology that appears most promising for the SOENs platform (Shainline et al., 2018, 2019). In these memory cells, circulating current persists indefinitely in a loop of superconducting wire. The current in the loop can be controlled by adding/removing magnetic-flux quanta with standard JJ circuitry. This memory loop is then inductively coupled to a wire supplying a bias current to a Josephson junction at the synapse (*J*_sf_ in Figure 2A). When the synaptic SNSPD detects a photon, the biased junction will add an integer number of fluxons to another integrating superconductive loop (analogous to the membrane capacitance of a neuron). The number of fluxons added to the integration loop is a function of the bias supplied to the JJ, which is determined by the magnitude of current circulating in the memory loop. The number of analog memory levels in the memory loop is determined by the inductance of the loop, which is easily set with the length of a wire. High-kinetic-inductance materials (Tolpygo et al., 2018) enable memory storage loops with over a thousand levels (10 bits) to be fabricated in an area of 5 μm × 5 μm.

The loop-memory approach has several strengths. The memory is nearly analog and updates are nearly linear. Memory is updated by the switching of a JJ, which involves only a change of the phase of the superconducting wave function. This phase can switch 10^11^ times in a second, so the endurance metric defined in the previous section is not an issue. This stands in contrast to room-temperature memories requiring material changes (filament formation, phase changes, etc.) which are often associated with degradation over time. Loop memory is also attractive from a fabrication perspective as it requires no additional materials or devices. The simplicity of the memory lends itself favorably to 3D integration, provided cross-talk from nearby loops can be mitigated. Plasticity circuits based on loop memories will also operate at the energy scale of single photons and flux quanta (10^−19^ J), which is commensurate with the rest of the circuitry in the network. This allows weight updates to be performed with the spikes the network produces in standard operation, reducing peripheral circuitry. There is no need to engineer differently shaped pulses for READ and WRITE operations, and the synapse does not need to be taken offline during programming. Simulations have demonstrated STDP learning with circuits containing four additional Josephson junctions (Shainline et al., 2019).

Two aspects of loop memory are concerning. First, loop memory is not strictly non-volatile. While circulating current can persist in a superconducting loop without any power supply, superconductivity must be maintained. If the temperature of the system is raised above the critical temperature of the superconducting material, the memory will be lost. Mechanisms for transferring weights stored in current loops to non-volatile solutions will need to be developed if the system's state is to be persevered upon reaching room-temperature (i.e., for maintenance or during a power interruption). The second weakness of loop memory is the size. The employed superconducting loops, as well as the transformers that couple them, will be large compared to all of the other solutions discussed. The consequences of these large-area components must be considered in the context of the entire system, which we discuss next.

## 5. System Level Considerations

Here we consider aspects concerning the integration of the components previously discussed and how systems may reach the scale of the brain. Basic graph theory metrics and the assumption of 300-mm fabrication processes allow us to assess area constraints and the benefits of 3D integration. It is found that at least five planes of photonic routing will be required in either platform to achieve brain-scale systems. Prospects for 3D integration of active elements are addressed. It also must be stressed that an optoelectronic system of the complexity of the human brain will be abjectly impossible on a single 300-mm wafer in either case. A possible vision for connecting many wafers is discussed. Finally, we analyze cooling and power concerns, finding that neither should preclude the development of brain-scale systems in either platform.

### 5.1. Considerations From Graph Theory

Neurons in brain regions active in cognition, such as the cerebral cortex and hippocampus, are characterized by a high degree of connectivity—often in excess of ten thousand connections per neuron (Braitenberg and Schuz, 1998; Buzsáki, 2006). These connections often extend across appreciable spatial distances. Creating and maintaining these connections comes with high metabolic and spatial costs. The severely constrained biological brain would not support such expenditures if they were not advantageous to cognition (Bullmore and Sporns, 2012).

One reason why such high connectivity is necessary relates to efficient communication across the network. Rapid communication can only be achieved if the average path length across the network is small. In the language of graph theory, a network is a collection of nodes connected by edges. To calculate the shortest average path length across the network, one calculates the number of edges that must be traversed to travel from one node to another node in the network. One takes the mean of this quantity over all pairs of nodes. The shortest average path length ($\bar{L}$) is a global metric that offers a glimpse at the efficiency with which information can be communicated across space.

Equation 6 provides the relationship between $\bar{L}$ and the number of edges connected to a node, or in our case, the number of synapses per neuron ($\bar{k}$) for a random network. In a random network, nearby and distant connections are equally probable. Specifically, the equation holds for Erdös-Rényi random graphs of networks with *N*_tot_ neurons (Fronczak et al., 2004):

(6) $$\begin{array}{l} \bar{k}=\text{exp}\left[\frac{\text{ln}\left(N_{\text{tot}}\right)-\gamma }{\bar{L}-1/2}\right], \end{array}$$

where γ ≈ 0.5772 is Euler's constant. For a network with 10^6^ neurons, each neuron must make nearly 10,000 connections to support an average path length of two, and 200 synapses must be formed to support a path length of three. For a network with 10^8^ neurons, more than 100,000 synapses are required for a path length of two, and more than 1,000 for a path length of three. The human hippocampus is a module with roughly 10^8^ neurons, each with 10,000–50,000 nearly spatially random connections. The objective of achieving an average path length between two and three may be an important reason why the hippocampus prioritizes this exceptional degree of connectivity (Buzsáki, 2006). The cerebral cortex in the human brain contains more than 10^10^ neurons, each with roughly 10,000 connections. This analysis indicates that a path length between two and three cannot be achieved across the entire cortex, and accordingly the cortex is constructed with a hierarchical, modular architecture (Simon, 1962; Meunier et al., 2010) with high connectivity and efficient communication within smaller modules, and more sparse connectivity between modules separated by larger distances (Mountcastle, 1997; Meunier et al., 2010; Bota et al., 2015; Betzel and Bassett, 2017).

While more sophisticated graph metrics can further elucidate the network concepts underlying cognition (Bullmore and Sporns, 2009), the simple, global metric of average shortest path length can help inform scaling analysis of artificial cognitive hardware at this early stage of development. We next consider the constraints $\bar{L}$ puts on the size of synaptic circuits.

### 5.2. Generic Spatial Constraints

Based on the significance of the interplay between the hippocampus and cerebral cortex in cognition (Friston and Buzsáki, 2016), we assume hardware for artificial neural systems will make use of similar architectural principles. Here we assume optoelectronic circuits will be fabricated using the conventional sequential, planar processing techniques of the silicon microelectronics industry. Photonic planes will implement the passive optical interconnects and electronic planes will accommodate all active electronics for neuronal function. We further specify to consideration of 300-mm wafers and seek a relationship between the network path length and the size of components on the wafer.

The area of a neuron occupied by its photonic waveguides can be approximated in a similar manner to the wires for electronic circuits (Keyes, 1982). This gives the following expression for the area of passive photonic circuitry:

(7) $$\begin{array}{l} A_{p}={\left(\frac{kw_{\text{wg}}}{p_{p}}\right)}^{2}. \end{array}$$

*p*_*p*_ is the number of photonic waveguide planes, *k* is the degree of each neuron (assumed identical), and *w*_wg_ is the pitch of waveguides. The area of a neuron due to electronic synaptic circuits is given by

(8) $$\begin{array}{l} A_{e}=\frac{kw_{\text{sy}}^{2}}{p_{e}}. \end{array}$$

**Figure 5:**
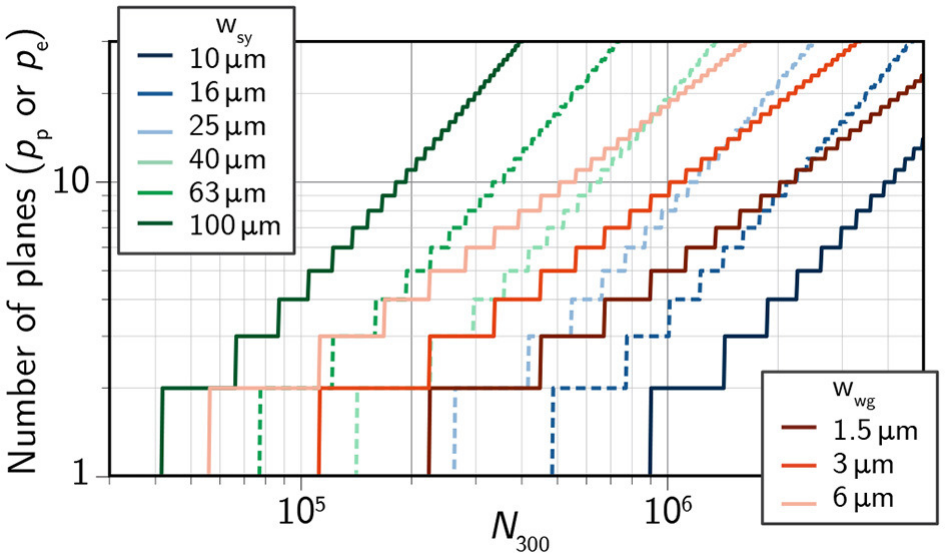
Number of planes of active synaptic circuits (*p*_*e*_) and passive photonic waveguides (*p*_*e*_) required to maintain a path length of 2.5 as a function of the number of neurons on a 300-mm wafer (*N*_300_).

*w*_sy_ is the width of a synapse and *p*_*e*_ is the number of planes of electronic circuits. Both *N*_tot_*A*_*p*_ and *N*_tot_*A*_*e*_ are subject to the area constraint of a 300- mm wafer. We use these relations to calculate the number of planes (electronic and photonic) that will be required to maintain a path length of 2.5 across a network of a given size (Figure 5). See Appendix C for analysis of path length dependence on *w*_sy_ and *w*_wg_. A specific routing scheme is analyzed in reference (Shainline et al., 2019). More than 10 million neurons (less than a mouse brain) on a single 300-mm wafer appears out of reach for any platform.

### 5.3. Fabrication Processes

We assume 300-mm silicon wafer processing. Wafer-scale integration has already been demonstrated for electronic neuromorphic systems (Schemmel et al., 2010). Still, even at this scale, reaching 10^6^ optoelectronic neurons per wafer is a tall order for either platform (Figure 5). We choose this integration metric somewhat arbitrarily; 10^6^ neurons per wafer corresponds to 10^4^ wafers for a human-cortex-scale system. This is roughly the same order as the number of processing units in modern supercomputers. If this target is to be reached, 3D integration at some level will be necessary. From Figure 5, it is clear that either platform will require a minimum of five photonic planes. Fortunately, photonic planes are quite amenable to 3D integration. Common waveguide materials include amorphous silicon (aSi), silicon nitride (SiN_*x*_) and silicon oxynitride (SiO_*x*_N_*y*_). These dielectric materials can be deposited at low temperature, enabling several multi-planar demonstrations (Sacher et al., 2015; Shang et al., 2015; Chiles et al., 2017; Zhang et al., 2018b). Additionally, low-temperature deposition makes such processes compatible with back-end CMOS fabrication. It should be noted that five photonic planes represents a best-case scenario, as wider waveguides have lower loss and only minimal reduction in average path length (Supplementary Information C).

3D integration of active electronics is less straightforward, particularly for the semiconductor approach. 3D CMOS integration has been the subject of decades of research (Rosenberg, 1983; Knickerbocker et al., 2008; Sakuma et al., 2008; Vinet et al., 2011; Lim, 2013; Zhao et al., 2015; Elfadel and Gerhard Fettweis, 2016; Li et al., 2017) and still faces uncertainty. Required high-temperature processing steps for dopant activation and contact anneals typically have a degrading effect on previous layers. Much of 3D integration of silicon microelectronics takes place at the die scale (Elfadel and Gerhard Fettweis, 2016), which is incommensurate with the scale of systems under consideration. For the semiconductor scenario, the best course of action may be to abandon 3D active electronics altogether in favor of simply reducing the footprint (*w*_sy_) of synapses. We see again from Figure 5 that nearly 10^6^ neurons can be integrated on a single plane if each synapse is on the order of 10 μm × 10 μm. This may be a challenging benchmark to reach with high-functionality synapses implementing complex plasticity and dynamics. Subthreshold circuits that have embraced larger CMOS nodes for decreased variability may need to adjust to more modern nodes, of which there is some precedent (Rubino et al., 2019). Additionally, photodetectors will be on the micron scale and long time-constant capacitors can require significant area (Appendix A) (Indiveri and Sandamirskaya, 2019). Both of these elements would however be fabricated on separate planes from MOSFETs.

Superconducting platforms would likely take the opposite approach, embracing 3D integration in the face of necessarily large device areas. Superconducting electronics, including active JJs, are routinely deposited at low temperatures (< 180 ℃). Integrated circuits with two stacked planes of JJs have been demonstrated by two research laboratories (Ando et al., 2017; Tolpygo et al., 2019), along with multiple of planes of SNSPDs (Verma et al., 2012). This is particularly important, as superconducting systems will not be able to reach 10^6^ neurons per wafer without 3D integration. A reasonable estimate for a superconducting synapse may be 30 μm on a side (Supplementary Information B). Such a size would require eight electronic planes.

We note that even if *p*_*p*_ = *p*_*e*_ = 1, it is still possible to fabricate wafers with 10^6^ neurons, provided $\bar{k}=100$, giving $\bar{L}=3.5$ (Figures 9, 10 in Supplementary Information C). While this does not match the short path lengths of cognitive circuits in the brain, such a network is likely to have significant technological and scientific utility while offering an intermediate-term practical objective.

### 5.4. Constructing Multi-Wafer Systems

Given that neither system will scale to billions of neurons on a single wafer, many wafers (~10,000) will need to be connected together to support human-brain-scale computing. A vision for a multi-wafer system is discussed in reference (Shainline, 2021) for the SOENs platform. Briefly, wafers are stacked and free-space optical communication is used to form highly inter-connected columns mimicking the modular structure of biological circuits (Mountcastle, 1978, 1997; Meunier et al., 2010; Bota et al., 2015; Betzel and Bassett, 2017). Columns are coupled to each other with lateral inter-wafer connections, but such connectivity is more sparse than that within a column. Optical fibers provide low-loss communication over long distances.

Achieving systems of this scale requires advances, particularly in wafer-scale circuit integration and system-level construction. A phenomenon akin to Moore's law, with ever-decreasing feature sizes enabling ever-higher integration density is unlikely to carry this concept forward, as many device sizes are limited by other physical considerations. Metrics related to number of planes of integrated circuits and number of wafers in a system may be more relevant to chart progress in neuromorphic supercomputing. Gradual progress may be possible by consistently scaling up, but it is difficult to envision this sustained trend without a powerful economic drive.

### 5.5. Power Consumption and Cooling

#### 5.5.1. Cooling Systems

Cooling systems will be a key component to either platform. For superconducting electronics, the system will fail completely if the temperature rises above the critical temperature (*T*_*c*_). Superconducting neuromorphic systems will rely on niobium (*T*_*c*_ = 9.3 K) or a material with a similarly low *T*_*c*_. Liquid helium (4.2 K) is the cryogen of choice for such temperatures. Cooling systems will add significantly to the power consumption of superconducting electronics. The power efficiency of a refrigeration system is measured by its specific power (Alekseev, 2015). The specific power gives the number of watts consumed by the refrigeration system for every watt of heat removed. The theoretical limit for specific power, given by the Carnot limit, is $\frac{T_{H}-T_{C}}{T_{C}}$. For liquid helium temperature (4.2 K), the Carnot limit demands that at least 74 watts of refrigeration power are required to remove every watt of heat produced on-chip if the system is operated in a 300 K ambient. State-of-the-art systems have reached specific powers below 400 W/W. Auspiciously, the most efficient refrigeration systems also tend to have the highest heat loads. The ability to cool heat loads as high as 10 kW at 4.2 K have already been demonstrated by commercially available systems (Holmes et al., 2013). Throughout this paper we assume a more conservative specific power of 1, 000 W/W, representative of the smaller scale cryogenic systems used in most laboratories today. It does not appear that cryogenic capability will be an insurmountable obstacle toward large-scale superconducting neural systems.

#### 5.5.2. Power Limitations

**Figure 6:**
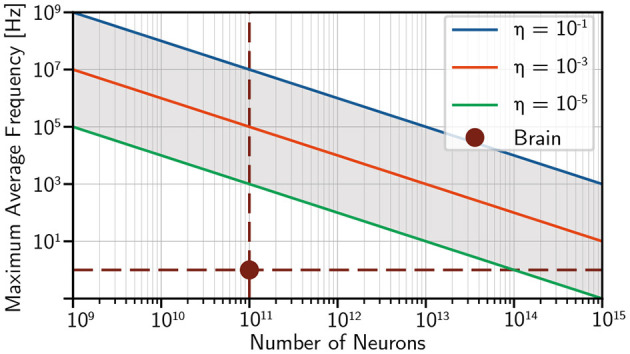
Tradeoff between size and average spiking frequency for a population of optoelectronic neurons with a power budget of 10 MW ($f=\eta *\frac{10\text{MW}}{\left(N_{\text{neurons}}^{*}N^{*}E_{opt}\right)}$. Fan-out (*N*) is 10^3^ and the optical energy needed at each synapse is assumed to be 1 fJ (accounting for cooling in superconductor case). This likely would correspond to the limits of either superconductor or semiconductor neurons.

Modern supercomputers typically consume megawatts of power. Tianhe 2, for instance, requires 17.8 MW for operation (and another 6.4 MW for cooling) (Tolpygo, 2016). If we thus assume a total power budget of 10 MW, we can analyze the trade-off between average firing rate and number of neurons. We assume 1 fJ of optical energy is required to initiate a firing event at each synapse and plot the maximum average frequency spiking frequency for several different optical link efficiencies in Figure 6.

Power does not appear to be a limiting factor in achieving brain-scale and brain-speed optoelectronic networks. If the power resources of modern supercomputers were dedicated to a brain-scale optoelectronic neuromorphic system, average spiking rates on the order of 10 kHz (10^4^ speedup over biology) appear feasible even with relatively inefficient optical links. Such a system may enable brain-scale computation with time accelerated by four orders of magnitude.

Another factor to consider is power density. There is a maximum power density that can be handled by heat removal systems for both the semiconducting and superconducting case. In the semiconductor case, high-performance computing routinely generates power densities of hundreds of watts per square centimeter (Tolpygo, 2016). A theoretical limit of around 1 kW/cm^2^ is postulated in Zhirnov et al. (2003). In contrast, superconducting systems will be required to operate at significantly lower power densities. Roughly 1 W/cm^2^ is a conservative limit for on-chip power density that can be cooled with liquid helium (Tolpygo, 2016). Superconducting optical links appear to be capable of dissipating about three orders of magnitude less energy per bit, approximately canceling out the limited power density requirements of superconducting systems in comparison with semiconductors. In practice, it might well be the case that mature, sophisticated synapses and neurons will occupy so much area that these power density limitations will be of no consequence. For instance, even with link efficiency of η = 10^−4^, a synapse would require a lateral dimension of less than 30 μm for power density considerations to limit spiking to less than 1 GHz. Section 5 argued that superconducting synapses are not likely to be smaller than this. 10 μm semiconducting synapses could reach 1 GHz with 1 × 10^−3^ efficiency. However, optoelectronic systems will have nonuniform power dissipation across the chip/wafer, with most of the power being dissipated at the light sources. A more in-depth analysis is required to see if heat removal will be an issue near the light sources in particular, but for the superconducting case it is convenient that the light sources themselves are not superconducting, and can afford to be raised to higher temperatures without failure. Concerns about local heating may be assuaged with layouts that sufficiently shield and/or separate thermally sensitive devices from the light sources.

## 6. Conclusion

**Figure 7:**
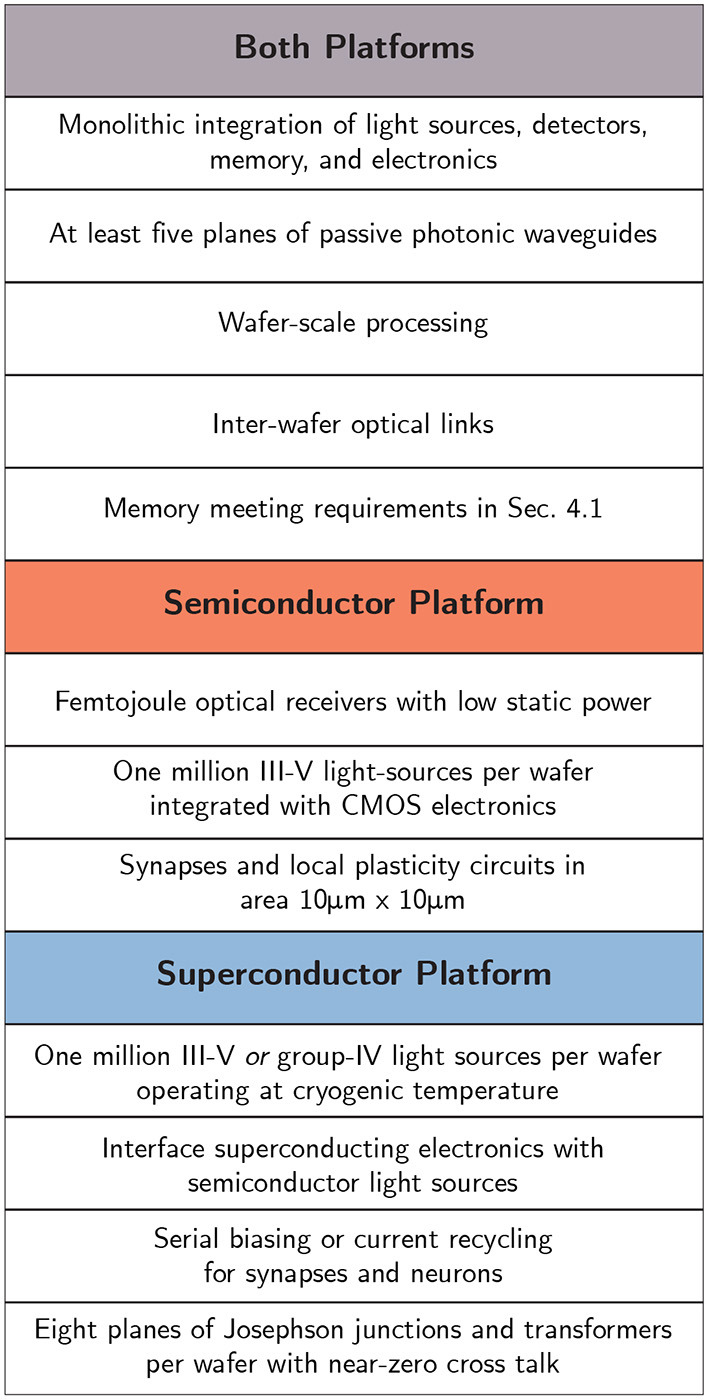
Summary of necessary hardware demonstrations for each platform if human-brain-scale artificial cognition is to be achieved.

The prospects of neuromorphic systems at the scale of the brain and beyond are tantalizing. The fan-out capability of optical communication coupled with the computational power of electronic circuitry makes optoelectronic systems a promising framework for realizing these high ambitions. However, there is no technology platform that is ready to support optoelectronic spiking networks of the scale and sophistication of the human brain. Making this vision a reality will require breakthroughs at the device level, no matter which path is chosen, particularly with regard to integrated light sources. Beyond that, several different classes of devices must be integrated alongside each other, which further reduces the likelihood for success. Efficient, densely integrated light sources, waveguide-coupled detectors, local memory devices, and capable neuronal circuitry all must be consolidated onto a single platform. Candidates for all requisite devices can be proposed for either semiconducting or superconducting platforms, and the two systems may be capable of similar performance. However, the technological paths toward achieving brain-scale systems with the two platforms diverge in important respects (Figure 7).

Semiconductor platforms hold advantages in technological maturity, room-temperature operation, and perhaps speed. Spike rates in excess of 10 GHz may be feasible, but only for systems significantly smaller than the human brain due to power constraints. Semiconductor receivers can potentially operate with extremely low energies per spiking event (sub femto-joule), making them a worthy competitor of superconducting single photon detectors. However, these low energy receivers require significant optical power from integrated light sources. To achieve biological-scale fan-out, either very bright light sources, repeatering schemes (costing area and yield), or additional gain stages (costing power) will need to be included. In terms of neuronal computation, semiconductor neurons have already demonstrated impressive functionality and low-power operation that should be capable of integration with optical communication infrastructure, provided the long-standing challenges with CMOS-integrated III-V light sources can be overcome. Synaptic memory is a major open question, but a variety of non-volatile memory solutions have seen extensive investigation, and time will tell if one technology can meet the requirements we have laid out for brain-scale optoelectronic systems. 3D integration of transistors, photodetectors, and memory may not be a feasible solution, meaning aggressive scaling of synaptic circuits while maintaining complex functionality is perhaps a better strategy. The fabrication processes for mature semiconductor neural systems may prove to be prohibitively complicated and heterogeneous, perhaps requiring different processing strategies for sources, detectors, and memories. If wafer-scale monolithic integration of these components cannot be achieved, and chip-scale die-stacking techniques are required, the outlook for achieving brain-scale systems is limited.

Superconducting optoelectronic neural systems suffer from a comparatively primitive fabrication ecosystem, but the incorporation of superconducting devices provides several intriguing properties. SNSPD receivers place nearly the theoretical minimum burden on integrated light sources. This attribute compounds positively with the improvements in efficiency for light sources operating at cryogenic temperatures. Integration of light sources with superconducting electronics does not appear to have the same material integration challenges as integration with CMOS, but this state of affairs may be due to the lack of attention the effort has received. These factors make the large-scale integration of light sources appear more tractable than in the semiconductor case—perhaps even opening the door to silicon as an active optical material. Driving these light sources with superconducting electronics, however, has yet to demonstrate the performance required for this application. The implementation of a high-impedance pulse-and-reset circuit remains an open challenge. For computation, superconducting neuronal circuits appear just as capable of implementing complex neuronal and synaptic behaviors as their CMOS counterparts, but will need to be designed with serial biasing in order to scale. Additionally, some speed advantages present in superconducting electronics will be negated by the response time of SNSPDs (<1 GHz). Of course, even if maximum spike rates are limited to 20 MHz, this would still represent a speed-up of four orders of magnitude over biological systems. Memory seems to be a strength for the superconducting platform, as superconductivity provides new avenues of storing synaptic weights. Loop memory in particular may be capable of implementing plasticity mechanisms that operate with only the signals produced through normal network activity. Caution is in order here, however, as superconducting synaptic plasticity mechanisms have scarcely been explored. 3D integration may yield more readily in the superconductor platform. The inconvenience of cryogenic cooling is a serious consideration, but power and heat removal estimations indicate this is unlikely to be a limiting factor for brain-scale systems. *If* all these issues can be resolved, superconducting optoelectronic systems may require simpler manufacturing processes than the semiconductor approach, as the material ecosystem could potentially be parsimonious. Of course, superconducting foundries are far less developed than their semiconductor counterparts, which may negate these advantages in the near-term.

We would be remiss to paint the quest for neuromorphic supercomputing as only a question of hardware. The inner workings of the brain are the subject of intense investigation, and the emergent phenomena of cognition and consciousness remain taunting, increasingly lonely enigmas entrenched in the netherworld between philosophy and science. Watershed breakthroughs in neuroscience and algorithmic development will be required for the discussed hardware platforms to have practical applications, although the hardware platforms themselves may be of use in helping to unravel some of these mysteries. The question of whether it is prudent to develop hardware before algorithms has pestered the field of neuromorphic computing since its inception. In this case, we believe that the length of development, rich opportunities for spin-off technologies, and inestimable potential make such hardware development well-worth pursuing even at this incipient stage.

## Data Availability Statement

The original contributions presented in the study are included in the article/Supplementary Material, further inquiries can be directed to the corresponding author/s.

## Author Contributions

All authors listed have made a substantial, direct and intellectual contribution to the work, and approved it for publication.

## Conflict of Interest

The authors declare that the research was conducted in the absence of any commercial or financial relationships that could be construed as a potential conflict of interest.

## Publisher's Note

All claims expressed in this article are solely those of the authors and do not necessarily represent those of their affiliated organizations, or those of the publisher, the editors and the reviewers. Any product that may be evaluated in this article, or claim that may be made by its manufacturer, is not guaranteed or endorsed by the publisher.
