# Supplementary material for: Considerations for Neuromorphic Supercomputing in Semiconducting and Superconducting Optoelectronic Hardware
Source: Front Neurosci. 2021 Sep 6;15:732368. doi: 10.3389/fnins.2021.732368 (PMC8450355; doi:10.3389/fnins.2021.732368)
Supplement: Supplementary file 1 [file Presentation_1.pdf]

Supplementary Information: Considerations for neuromorphic supercomputing in semiconducting and superconducting optoelectronic hardware

Bryce A. Primavera and Jeffrey M. Shainline

April 2021

A Implementing Long Time Constants

For the DPI synapse (section 3) the time constant is given by [1]:

$$\tau = \frac{C_{\text{si}} V_{\text{th}}}{\kappa I_\tau}, \tag{A.1}$$

where I_τ is the current leaking off of the capacitor, set with transistor T_3, V_{th} is the thermal voltage, and κ is the sub-threshold slope factor (typically order 1). Operating in the subthreshold regime allows I_τ to be reduced to femtoamps [2]. Metal Insulator Metal (MIM) capacitors ultizing high-k dielectrics can reach capacitance densities around 20 fF/µm^2 [3]. The maximum achievable time constant as a function of synapse width is shown in figure 8 for $I_\tau = 10\,\text{fA}$, $\kappa = 1$, and $V_{\text{th}} = 25\,\text{mV}$. Since MIM capacitors can be fabricated on a separate layer from transistors, the entire 10 µm × 10 µm area per synapse target identified in Sec. 5 could be dedicated to capacitor area.

For SOENs synapses, $L_{\text{si}}/r_{\text{si}}$ sets the time constant. Inductors and resistors will most likely be fabricated on separate layers, again conserving space. A meandering geometry gives the maximum inductance L_{si}, that can be fabricated in an area w_{sy}^2 as:

$$L_{\text{si}} = \frac{w_{sy}^2 L_\square}{w_{\text{wire}}(w_{\text{wire}} + w_{\text{gap}})}, \tag{A.2}$$

where $L_\square$ is the inductance per square of the material, and w_{wire} and w_{gap} are the minimum feature sizes. Small value resistors are fabricated by putting many wide resistors in parallel. The smallest (nonzero of course) resistor that can be fabricated in an area of w_{sy}^2 is then:

$$r_{\text{si}} = \frac{R_s w_{\text{gap}}(w_{\text{wire}} + w_{\text{gap}})}{w_{sy}^2}, \tag{A.3}$$

where R_s is the sheet resistance. The maximum time constant (τ_{max}) in area w_{sy}^2 is given by $L_{\text{si}}/r_{\text{si}}$:

$$\tau_{\text{max}} = \frac{w_{sy}^4 L_\square}{R_s w_{\text{wire}} w_{\text{gap}}(w_{\text{wire}} + w_{\text{gap}})^2} \tag{A.4}$$

The maximum achievable time constants as a function of synapse width is plotted in figure 8 for $R_s = .001\,\Omega/\square$ (corresponds to a 200 nm gold layer with resistivity of $2 \times 10^{-10}\,\Omega\cdot\text{m}$ at 4 K [4]), $w_{\text{wire}} = w_{\text{gap}} = 100\,\text{nm}$, and $L_\square = 160\,\text{pH}/\square$, corresponding to MoSi. For the large synaptic areas expected to be available to superconducting synapses via 3D integration, the superconducting approach can support significantly larger time constants than the semiconducting case. In Ref. 5, it is suggested that the human brain itself is limited by the maximum achievable time constant, and relies on network dynamics for certain types of long-term memory. The ramifications of hardware with time-constants far greater than biology are intriguing.

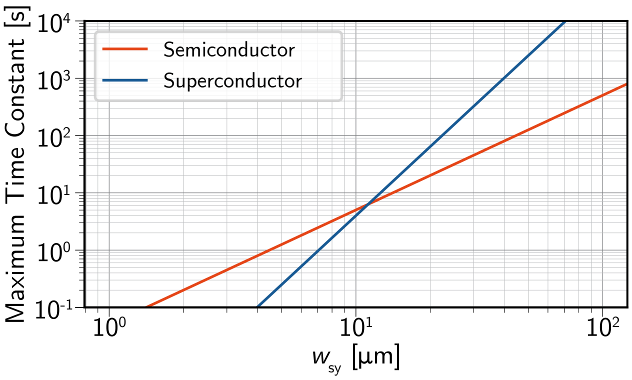

Figure 8: Maximum achievable time constant as a function of synapse width.

B Area and Energy in Superconducting Synapses

JJs will never be as small as MOSFETs [6], but the area of synaptic circuits is not limited by JJs. Large inductors/transformers that couple flux from storage loops to receiving SQUIDs are likely to be the components that consume the most area. The size of these components is determined by the critical current of the junctions used

in the SQUIDs (I_c), the permeability of free space (μ_0), SQUID inductance (L), and the magnetic flux quantum (Φ_0). Based on the typical SQUID design criterion [7] we expect $2LI_c/\Phi_0 = 1$. For a simple washer-type inductor geometry, $L \approx 1.25\mu_0 w_{\text{sq}}$, where w_{sq} is the inner dimension of the hole [8]. The energy, E_{sq} to produce two fluxons is approximately $2I_c\Phi_0$ for an appropriately biased junction. There is thus a trade-off between the size of a SQUID and its energy consumption:

$$w_{\text{sq}} \approx \frac{\Phi_0^2}{1.25\mu_0 E_{\text{sq}}} \tag{B.1}$$

I_c is further constrained by noise and the ability to interface with SNSPDs. $I_c = 300\,\mu\text{A}$ is reasonable and would correspond to $w_{\text{sq}} \approx 2.2\,\mu\text{m}$ and $E_{\text{sq}} \approx 1.2\,\text{aJ}$. If the optical energy per synapse is around 100 aJ for a 1% efficient link, about 170 fluxons can be produced per synapse event without dominating the power budget. In practice, each SQUID may require a lateral dimension about five times larger than w_{sq} to account for washer width, wiring, and spacing to minimize cross-talk. Additionally, each synapse will likely be composed of three or four SQUIDs—one for synaptic integration, one for loop memory, and perhaps two more for various plasticity functions. A reasonable estimate for synaptic size is then about $30\,\mu\text{m} \times 30\,\mu\text{m}$.

C Further Scaling Analysis

Figures 9 and 10 provide insight into how network connectivity constrains hardware for any planar fully-dedicated system. Figure 9 plots Eq. 6, giving the average node degree (number of synapses per neuron) necessary to maintain a given path length as a function of network size.

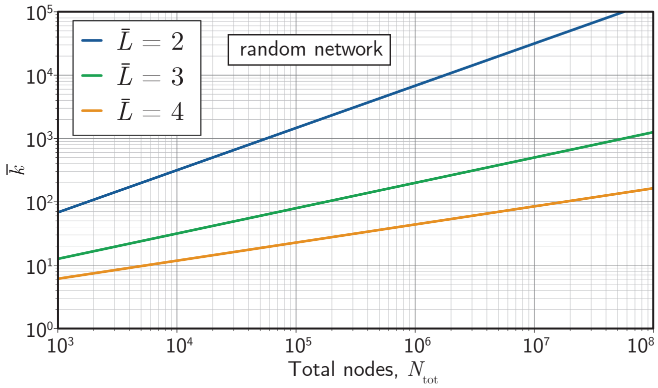

Figure 9: Average node degree as a function of network size for different path lengths.

Figure 10 plots the minimum achievable path length as function of synaptic width and waveguide width (Eqs. 8 and 7). Path length is relatively insensitive to waveguide pitch, suggesting that wider, and therefore lower loss waveguides may be beneficial. In contrast, we see that the synaptic size (w_{sy}) can be a major impediment to achieving low path lengths.

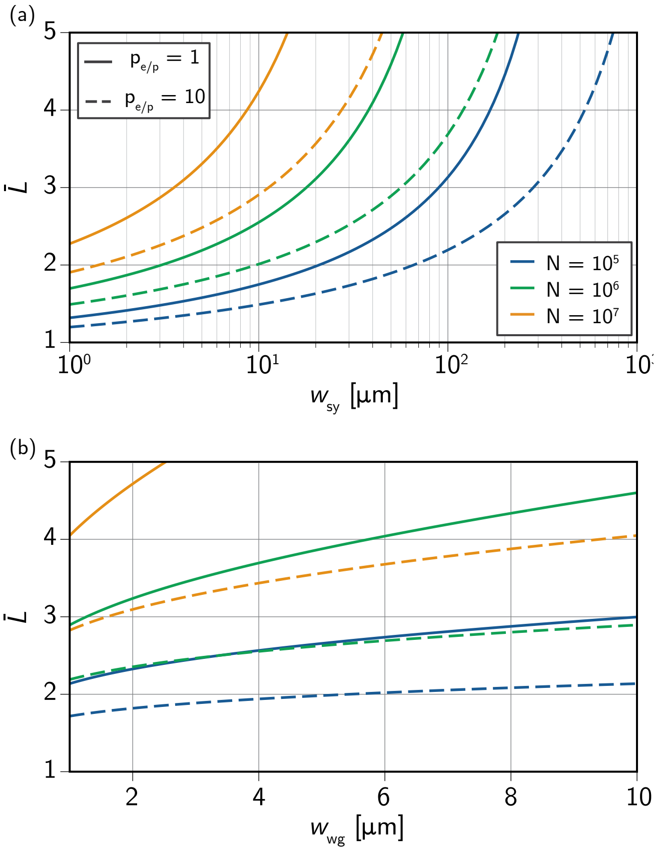

Figure 10: Path length versus feature size. (a) Path length versus width of electronic synapses. (b) Path length versus waveguide pitch. In both figures the solid lines correspond to a single plane of synapses or waveguides, and the dashed lines correspond to 10 planes of synapses or waveguides. The blue, green, and yellow traces correspond to the cases of 10^5, 10^6, and 10^7 neurons per 300-mm wafer, respectively.

References

[1] Elisabetta Chicca, Fabio Stefanini, Chiara Bartolozzi, and Giacomo Indiveri. Neuromorphic electronic circuits for building autonomous cognitive systems. Proceedings of the IEEE, 102(9):1367–1388, 2014.

[2] Bernabé Linares-Barranco and Teresa Serrano-Gotarredona. On the design and characterization of femtoampere current-mode circuits. ieee journal of solid-state circuits, 38(8):1353–1363, 2003.

[3] Yung-Hsien Wu, Bo-Yu Chen, Lun-Lun Chen, Jia-Rong Wu, and Min-Lin Wu. Metal-insulator-metal capacitor with high capacitance density and low leakage current using zrtio 4 film. Applied Physics Letters, 95(11):113502, 2009.

[4] Richard Allen Matula. Electrical resistivity of copper, gold, palladium, and silver. Journal of Physical and Chemical Reference Data, 8(4):1147–1298, 1979.

[5] Giacomo Indiveri and Yulia Sandamirskaya. The importance of space and time for signal processing in neuromorphic agents: the challenge of developing low-power, autonomous agents that interact with the environment. IEEE Signal Processing Magazine, 36(6):16–28, 2019.

[6] Sergey K Tolpygo. Superconductor digital electronics: Scalability and energy efficiency issues. Low Temperature Physics, 42(5):361–379, 2016.

[7] J. Clarke and A.I. Braginski, editors. The SQUID handbook. Wiley-VCH.

[8] J Jaycox and M Ketchen. Planar coupling scheme for ultra low noise dc squids. IEEE Transactions on Magnetics, 17(1):400–403, 1981.
